# Discontinuation of reimbursement of benzodiazepines in the Netherlands: does it make a difference?

**DOI:** 10.1186/1471-2296-13-111

**Published:** 2012-11-21

**Authors:** Boudewijn J Kollen, Willem Jan van der Veen, Feikje Groenhof, Gé A Donker, Klaas van der Meer

**Affiliations:** 1 Department of General Practice, University Medical Centre Groningen, University of Groningen, Ant. Deusinglaan 1, Groningen, 9713 AV, The Netherlands; 2 Municipal Health Services Drenthe, Assen, The Netherlands; 3 Health Centre De Weide, Hoogeveen, The Netherlands

## Abstract

**Background:**

In an attempt to control chronic benzodiazepine use and its costs in the Netherlands, health care insurance reimbursement of this medication was stopped on January 1^st^ 2009. This study investigates whether benzodiazepine prescriptions issued by general practitioners changed during the first two years following implementation of this regulation.

**Methods:**

Registry study based on data from all benzodiazepine users derived from the Registration Network Groningen. This general practice-based research network collects longitudinal data on the primary care administered to about 30,000 patients. Based on the number of quarterly accumulated prescription days, a comparison was made of benzodiazepine prescriptions issued between 2007/2008 and 2009/2010. Also investigated was which type of user (i.e. short-term or long-term) showed the most change.

**Results:**

Information on benzodiazepine prescriptions among 5,200 patients from 16 consecutive trimesters between 2007 and 2010 was available for analysis. A significant reduction in prescription days was observed between 2007/2008 and 2009/2010. Overall, an estimated 1.73 (CI:-1.94 to -1.53; *p<*0.001) days were less prescribed per trimester after the termination of reimbursement. In particular, short-term users experienced a reduction in prescription days in 2009 and 2010. The number of long-term users decreased by 2.3%, while the number of individuals that did not use increased by 4.2%.

**Conclusions:**

A total reduction of almost 14 prescription days was observed over eight trimesters after implementation of the regulation to terminate the reimbursement of benzodiazepines. Short-term users were mainly responsible for this reduction in prescription days in 2009 and 2010. Although long-term users did not alter their benzodiazepine use in 2009 and 2010, the number of long-term users decreased slightly.

## Background

Benzodiazepines are frequently prescribed to patients suffering from anxiety, nervousness or sleep problems. In 2007, over 10 million prescriptions of benzodiazepines for 1.8 million individuals were issued in the Netherlands. The general practitioner (GP) is responsible for most of these prescriptions [1].

Although it is generally recommended to use benzodiazepines for only a brief period, a substantial proportion of patients become long-term chronic users of this medication [2]. Long-term use carries the risk of dependence, cognitive impairment, accidents and falls, especially in the elderly [3]. In the Dutch population, this long-term use of benzodiazepines has remained persistent over the years (1992–2002) despite efforts to reduce its use and renewal of the guidelines [4]. Even attempts by community pharmacies in collaboration with GPs to substantially reduce the long-term use of benzodiazepines failed to succeed [5].

In the Netherlands, in an attempt to impose a reduction in benzodiazepine use, health care insurance reimbursement of this medication was stopped on January 1^st^ 2009. Although this regulation was primarily implemented to reduce or prevent the long-term use and addictive effects of this medication, it also served to diminish the costs involved in supplying this medication. However, apart from long-term users, also first and short-term users were affected by this regulation.

Not all patients were deprived of reimbursement for this medication. The administration of benzodiazepines remained funded for individuals who are dependent on this medication in the absence of an adequate alternative. This applies to patients that use benzodiazepines to curtail epileptic seizures, to individuals with anxiety disorders in which at least two anti-depressants fail to ameliorate symptoms, and to patients with multiple psychiatric conditions that necessitate the administration of high doses of benzodiazepines. Finally, palliative sedation used in terminal care is also exempted from this regulation, as are patients that require diazepam medication for muscular spasms resulting from neurological disorders.

In preparing for implementation of this regulation, physicians and pharmacists were asked to inform their patients that reimbursement of this medication would be terminated in 2009 and that those who continue to use benzodiazepines would bear the costs of purchasing the medication. Physicians were encouraged to discuss discontinuation with their patients as a viable option and offer suitable alternatives. Discontinuation of this medication would then best be carried out gradually in close collaboration with the physician and pharmacist.

This reimbursement restriction has led to a moderate decrease in the number of incident diagnoses and initiation of benzodiazepine use in patients with newly diagnosed anxiety or sleeping disorders in 2009 [6]. However, whether the overall use (prevalence) has changed over a longer period remains unknown.

In the present study, we investigated whether benzodiazepine use changed after termination of the reimbursement of the medication. The number of quarterly accumulated prescription days for each benzodiazepine user was compared between the 2007/2008 and 2009/2010 periods. Prescription days were defined as the number of days that benzodiazepines were prescribed during each trimester.

We anticipated a reduction in prescription days during 2009 and 2010. In order to explore which type of user experienced the most change, the users were categorized into initial, short-term and long-term users. We hypothesized that long-term users would show the most change.

## Methods

Data from all benzodiazepine users in the Registration Network Groningen (RNG) were obtained. This general practice-based research network was established in 1989 and consists, at present, of three large group practices situated in the north-eastern part of the Netherlands. Participating GPs (n=20) register all their primary care. In this way, the annual care of about 30,000 regular patients is recorded longitudinally. All consultations, with the reasons for encounter as well as diagnoses and prescriptions, are recorded. Morbidity data are also electronically registered using the International Classification of Primary Care (ICPC) and each prescribed medication is fitted with an ICPC-based code. Prescriptions are automatically classified with an Anatomical Therapeutic Chemical (ATC) code. The benzodiazepines that are affected by the regulation are classified in the benzodiazepines derivatives category (ATC N05BA and N05CD) and benzodiazepines-related drugs (ATC N05CF).

This study was approved by the review board of the University Medical Centre of Groningen.

### Data analyses

The characteristics of patients from a particular GP and their observations usually differ slightly from those of patients from other GPs. As a consequence, GP-related patient information is to some degree clustered. The analysis of such data requires the implementation of multilevel statistical methods that adjust for this clustering.

Linear multilevel analysis (MLA) was conducted using MLwiN 2.23 to determine whether a change occurred in the number of prescription days. Multilevel models are hierarchical systems that estimate regression coefficients and their related variance components while at the same time adjust for the dependency of observations. The first level was defined as observation, the second level as patient, and the third level as GP. The iterative generalized least squares algorithm was used to estimate the regression coefficients and the likelihood ratio test to evaluate the necessity for allowing random intercept and regression coefficients into the model, while the Wald test was used to obtain a *p* value for each regression coefficient [7]. Outcome scores were plotted to check for compliance with model assumptions. For all tests, a two-tailed significance level of *p*<0.05 was used.

In addition, to investigate whether the changes in benzodiazepine consumption after termination of reimbursement depended on the type of user, users were categorized based on their number of quarterly registered prescription days. We identified initial users (individuals who did not use in the trimester prior to starting this medication), short-term users (individuals with less than 60 prescription days in a particular quarter), long-term users (individuals with 60 or more prescription days), quitters (individuals who were users in the trimester prior to discontinuing this medication), and a category unknown adapted from [1]. When no information was available in the preceding quarter, initial users and quitters could not be identified and were excluded from the analysis.

Subsequently, for each specific type of user interaction terms were fitted in the multilevel regression model to investigate whether the number of prescription days was dependent on the period of registration (2007/2008 versus 2009/2010).

In this analysis, an initial user could not also be classified as short or long-term user, even though being an initial and short or long-term user is not mutually exclusive. Because this compatibility problem affects short-term and long-term rates, a supplementary analysis was conducted to determine the real contribution of short-term and long-term users to the change in prescription rates before and after implementation of the regulation. In this analysis we used all information on short-term and long-term users, thus ignoring available information on initial users and quitters. In other words, the number of prescription days was compared between 2007/2008 and 2009/2010 for all short-term and long-term users categorized independently of the information in the preceding quarter.

## Results

Information on benzodiazepine prescriptions in 5,200 patients over a 4-year period (2007–2010) and subdivided into 16 trimesters was available for analysis. Of all users, 63% was female (n=3,259) and 37% male (n=1,941). The mean age during this study period was 53 (±18.8) years; 54 (±19.5) for females and 52 (±17.4) years for males. The percentage of missing values of the outcome variable, i.e. number of prescription days, was 10.6% (8.5% in 2007/2008 and 12.7% in 2009/2010). On average, benzodiazepines were prescribed during each trimester for 15.57 days in 2007/2008 and 13.45 days in the 2009/2010 (Table 1). Table 1 also presents the number of each type of user for each trimester in this open cohort.

**Table 1:** Mean number of prescription days and (mean) number of type of users between 2007 and 2010

	**Trimesters**																		
	**2007**				**2008**				***Mean (%)***	**2009**				**2010**				***Mean (%)***	***Difference means (p)***
	**1**	**2**	**3**	**4**	**1**	**2**	**3**	**4**		**1**	**2**	**3**	**4**	**1**	**2**	**3**	**4**		
Mean number of prescription days	12.98ǂ	15.45	15.65	15.94	15.51	16.14	15.98	16.90	***15.57***	13.34	13.03	13.05	13.38	13.23	13.56	13.61	14.48	***13.46***	***2.11***
*Type of user*																			
no user	ǂ	2642	2614	2608	2586	2619	2623	2532	***2603 (56.2)***	2597	2698	2802	2704	2645	2653	2642	2541	***2660 (60.0)***	***-56.82 (<0.001)***
initial	ǂ	458	404	449	417	399	389	435	***422 (9.1)***	270	365	363	429	378	367	351	408	***366 (8.3)***	***55.20 (<0.001)***
short-term	ǂ	612	604	624	636	620	642	662	***629 (13.6)***	762	513	518	521	559	514	533	509	***554 (12.5)***	***74.95 (<0.001)***
long-term	ǂ	556	591	580	580	594	592	606	***586 (12.7)***	454	461	445	447	445	474	467	483	***460 (10.4)***	***126.07 (<0.001)***
Quitter	ǂ	365	427	385	431	404	364	345	***389 (8.4)***	475	496	357	340	390	378	342	340	***390 (8.8)***	***-1.04 (<0.046)***
*Total*	ǂ	*4633*	*4640*	*4646*	*4650*	*4636*	*4610*	*4580*	***4629 (100)***	*4558*	*4533*	*4485*	*4441*	*4417*	*4386*	*4335*	*4281*	***4430 (100)***	
*Supplementary analysis of short vs. long term users*																			
short-term	1131	1100	1031	1109	1089	1057	1073	1138	***1091***	1071	904	909	981	972	916	922	949	***953***	***138***
long-term	459	583	615	598	602	628	617	626	***591***	467	478	465	459	460	489	483	509	***476***	***114.75***
*Total*	*1590*	*1683*	*1646*	*1707*	*1691*	*1685*	*1690*	*1764*	***1682***	*1538*	*1382*	*1374*	*1440*	*1432*	*1405*	*1405*	*1458*	***1429***	

ǂ The first trimester of 2007 is omitted due to or affected by the unavailability of information of prescription days from the preceding trimester. Because of this, no information on initial users and quitters was available for the first trimester of 2007. As a consequence, presenting information on short-term and long-term users would generate an overestimation when compared to the information on short-term and long-term users in the following trimesters. Mean is based on the trimester means of the preceding two years.

Multilevel analysis revealed a significant reduction in prescription days between 2007/2008 and 2009/2010. On average, compared to before implementation, there was an estimated 1.73 (CI:-1.94 to -1.53; *p<*0.001) reduction in prescription days during each trimester after implementation of the regulation. This reduction can be explained by changes occurring at all three levels, i.e. within (*p*<0.001) and between patients (*p*<0.001) and between GPs (*p*=0.02). The explained variance of this association is 0.4%.

No differences in gender (*p*=0.38) or person-time (*p*=0.28) before and after stopping reimbursement was found. This indicates that in this dynamic cohort person-time did not accrue differently between study periods as a result of dynamic inclusion and attrition rates.

**Figure 1:**
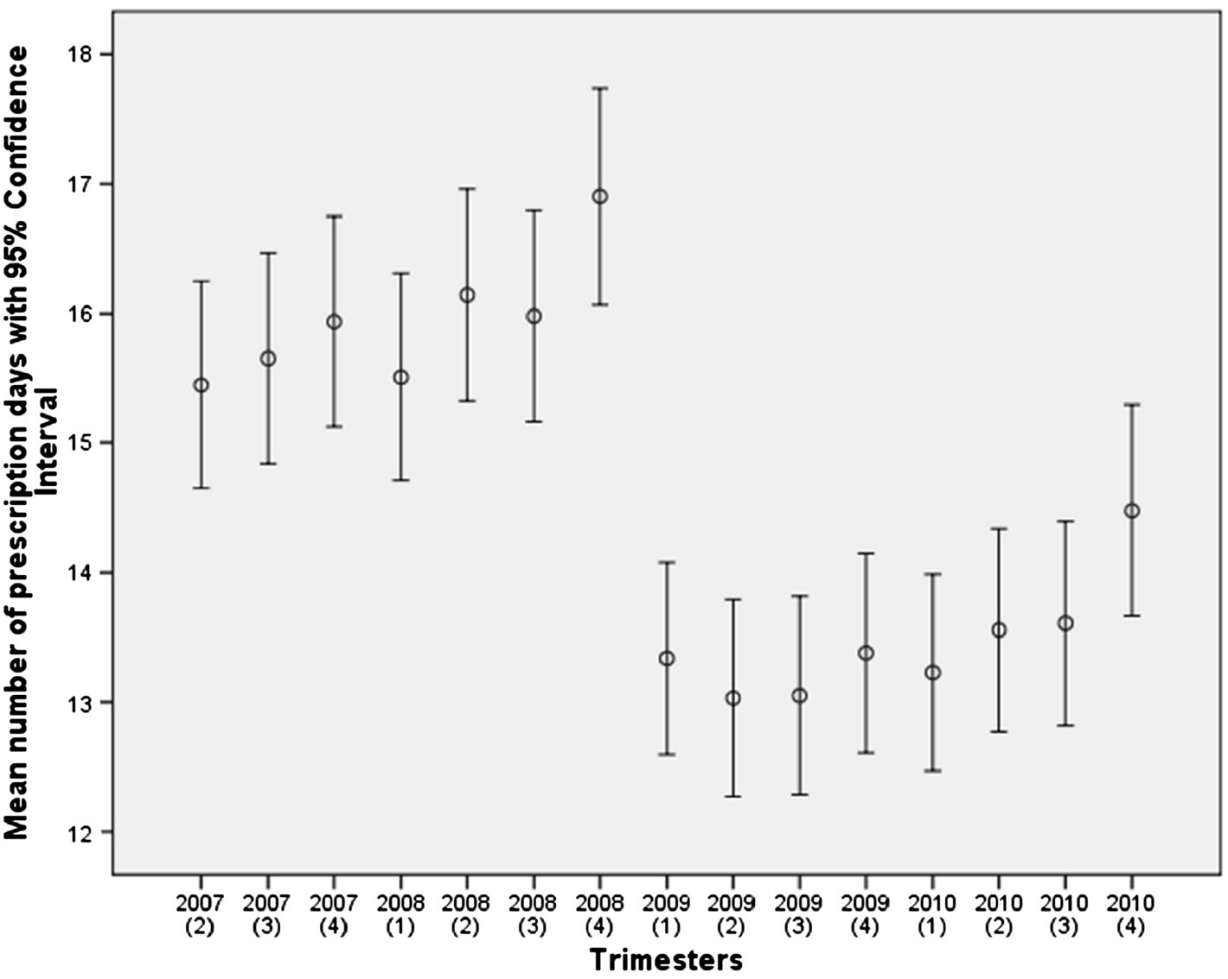
**Mean number of prescription days of benzodiazepines for each trimester between 2007 and 2010 and corresponding confidence interval.** The first trimester of 2007 is omitted due to the unavailability of information of prescription days from the preceding trimester.

Across all trimesters, a pattern is visible of increasing use of benzodiazepines towards the end of each year and towards the end of the 2007/2008 and 2009/2010 periods, with a sharp decline in prescription days between the last quarter of 2008 and first quarter of 2009 (Figure 1).

Overall, in 2009/2010 the mean number of individuals that did not use benzodiazepines (i.e. no users and quitters) had increased by 4.2% compared with the 2007/2008 levels, while the mean number of long-term users decreased by 2.3%. The mean number of initial users and short-term users showed a marginal decrease of 0.8% and 1.1%, respectively (Table 1).

At a benzodiazepine user and non-user level, the initial users, short and long-term users all showed less prescription days after implementation of the regulation. On average, long-term users experienced the greatest reduction (i.e. 1.4 days; *p<*0.001), while short-term users showed a reduction of 1.1 days (*p<*0.001) and initial users less than 1 day (*p=*0.001) per trimester (Table 2). However, when considering all available information on short-term and long-term users (some of whom were identified as initial users in the previous analysis) we found that between 2007/2008 and 2009/2010 the number of prescription days decreased significantly from 19.88 to 18.96 days during each trimester (-0.91, CI:-1.17 to -0.66; *p<*0.001) in short-term users. However, no significant decrease in benzodiazepine use was observed in long-term users (-0.14, CI:-0.48 to 0.20; *p*=0.41). In this supplementary analysis, almost all initial users are classified as short-term users (97.5% in 2007/2008 and 98.3% in 2009/2010) and the reduction in prescription days for initial users is added mainly to that of the short-term users. The absolute difference in number of prescription days per trimester between short-term and long-term users increased by almost 1 day (0.96, CI:0.33 to 1.58; *p*=0.003) from 47.63 prescription days in 2007 and 2008 to 48.59 days in 2009 and 2010.

**Table 2:** Mean number of estimated quarterly prescription days for type of user before and after the termination of reimbursement of benzodiazepines

**Type of user**	**2007-2008**	**2009-2010**	**Mean difference (CI)**	***p***
initial	16.98	16.31	-0.68 (-1.07–-0.28)	0.001
short-term	25.08	24.02	-1.05 (-1.39–-0.71)	<0.001
long-term	74.54	73.15	-1.40 (-1.75–-1.04)	<0.001

CI=confidence interval.

## Discussion

This study demonstrates a significant decrease of 1.7 days in the number of prescription days per trimester between 2007/2008 and 2009/2010. Although it is likely that the termination of reimbursement is partly responsible for this reduction in prescription days, the present study design is not suitable to establish a causal effect. Moreover, given the limited explained variance of the association, other factors have also contributed to this change. Furthermore, based on the magnitude of the large number of benzodiazepine users, significance is relatively easy to obtain in a sample of this size. More importantly, should this significant reduction in prescription days also be considered a relevant reduction? This is debatable as this reduction represents about 10% of the mean number of prescription days per trimester in the 2007/2008 period. Moreover, the observed increase in prescription rates towards the end of 2008 may be partially due to advanced prescribing of the GPs to assist their patients through the first months of 2009. As a consequence, the ensuing sharp decline in prescription rate at the beginning of 2009 is likely to be more pronounced. In the literature more dramatic reductions in prescription rates have been reported of up to 60% over a one-year period following their regulation [8]. In our study, the prescription rate started to rebound somewhat during the second year following the regulation. Therefore, a more realistic consideration of the long-term effects of the regulation to curtail the prescription rate of benzodiazepines can probably be attained over a prolonged period (e.g. 5 years).

Although this medication is relatively inexpensive to purchase, the economic gain as a result of this reduction may still be substantial given its widespread use. An upward trend can be observed in 2009 and 2010, especially towards the end of the registration period (Figure 1). It is not inconceivable that the number of prescription days for benzodiazepine may return to the 2008 level in the coming years.

**Figure 2:**
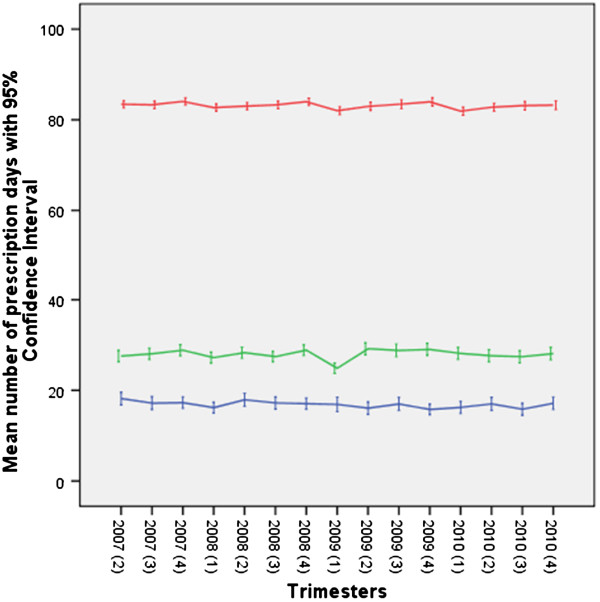
**Course of quarterly accumulated prescription days of benzodiazepines for long-term users (upper line), short-term users (middle line) and initial users (lower line) between 2007 and 2010.** The first trimester of 2007 is omitted due to the unavailability of information of prescription days from the preceding trimester.

As demonstrated in this study, initial users, short-term and long-term users contributed proportionally to this reduction in prescription days. Unlike long-term users and initial users, short-term users experienced a brief dip in their mean prescription days between the last quarter of 2008 and the second quarter of 2009 (Figure 2). Overall, the reduction in prescription days coincided with a reduction in the number of users, i.e. the number of individuals that did not use increased and the number of long-term users decreased.

However, when initial users are considered as either short-term or long-term users, a different picture emerges in that the reduction in prescription days in 2009/2010 is less pronounced and was only observed among short-term users. The number of prescription days among long-term users showed no significant change. Therefore, the primary objective of this regulation to substantially reduce long-term use may not have materialized.

### Limitations

In this study, no effort was made to identify substitution effects. These effects occur when subscriptions are issued for psychopharmacological drugs other than benzodiazepines. This, in turn, could have biased our outcome as current no users in our population may have been users of these other drugs. This effect was observed in the study of Weintraub et al. who investigated the consequences of the 1989 New York State triplicate benzodiazepine prescription regulations [8]. In addition to a decrease in the number of prescriptions for benzodiazepines, they reported a negative impact of the regulations, i.e. an increase in prescriptions for alternative medications [8]. Moreover, in our open cohort, inclusion and attrition rates of patients varied during the course of the study; this resulted in absent registrations in the general practice-based research network and missing values in the dataset. Whether individuals with missing values used benzodiazepines prescribed elsewhere is uncertain. Also, in the present study, benzodiazepine “use” was used as an approximation for benzodiazepine “prescriptions”. However, this may not always be accurate as in fact not all prescriptions of benzodiazepines may have led to their consumption. Similarly, in our study we assumed that “mean days of prescription” corresponds with the mean number of pills consumed; this may not be accurate. Furthermore, we did not define the assumed average maintenance dose per day for the benzodiazepines prescribed in the present study. As a result, we cannot rule out that attempts to lower the dose may have played a role in the decrease of the prescription days described as defined daily dose.

Finally, information on the first quarter of 2007 was incomplete because the effects of prescriptions issued in the last quarter of 2006 are missed in our analysis. Therefore, the estimated decrease of 1.7 days in the number of prescription days per trimester between 2007/2008 and 2009/2010 may represent a slight underestimation.

## Conclusions

A total reduction of almost 14 prescription days (representing 1.73 days over 8 trimesters) was observed over a two-year period after implementation of the regulation to terminate the reimbursement of benzodiazepines. Short-term users were mainly responsible for this reduction in prescription days in 2009 and 2010. Although long-term users did not alter their benzodiazepine use in 2009 and 2010, the number of long-term users decreased slightly.

## Competing interest

No support from any organization for the submitted work; no financial relationships with any organizations that might have an interest in the submitted work in the previous three years, no other relationships or activities that could appear to have influenced the submitted work.

## Authors’ contributions

BJK designed and performed data analyses and wrote the draft, WJvdV designed the study and assisted in draft writing and outcome interpretation, FG was responsible for data collection and management and assisted in outcome interpretation, GD assisted in draft writing and outcome interpretation, KvdM assisted in outcome interpretation. All authors approved the final draft.

## Pre-publication history

The pre-publication history for this paper can be accessed here:

http://www.biomedcentral.com/1471-2296/13/111/prepub
